# Immuno-Surgical Management of Pancreatic Cancer with Analysis of Cancer Exosomes

**DOI:** 10.3390/cells9071645

**Published:** 2020-07-09

**Authors:** Yu Takeda, Shogo Kobayashi, Masatoshi Kitakaze, Daisaku Yamada, Hirofumi Akita, Ayumu Asai, Masamitsu Konno, Takahiro Arai, Toru Kitagawa, Ken Ofusa, Masami Yabumoto, Takaaki Hirotsu, Andrea Vecchione, Masateru Taniguchi, Yuichiro Doki, Hidetoshi Eguchi, Hideshi Ishii

**Affiliations:** 1 Center of Medical Innovation and Translational Research (CoMIT), Osaka University Graduate School of Medicine, Suita, Yamadaoka 2-2, Osaka 565-0871, Japan; ytakeda26@gesurg.med.osaka-u.ac.jp (Y.T.); momokitakaze@gmail.com (M.K.); aasai@cfs.med.osaka-u.ac.jp (A.A.); mkonno@cfs.med.osaka-u.ac.jp (M.K.); t.arai@unitech-op.com (T.A.); toru@kyowakai.com (T.K.); oof21443@ideacon.co.jp (K.O.); yabumoto.masami@gmail.com (M.Y.); hirotsu@hbio.jp (T.H.); ydoki@gesurg.med.osaka-u.ac.jp (Y.D.); heguchi@gesurg.med.osaka-u.ac.jp (H.E.); 2 Department of Gastroenterological Surgery, Graduate School of Medicine, Osaka University, Suita 565-0871, Japan; s-kobayashi@umin.ac.jp (S.K.); dyamada@gesurg.med.osaka-u.ac.jp (D.Y.); hakita@gesurg.med.osaka-u.ac.jp (H.A.); 3 Artificial Intelligence Research Center, The Institute of Scientific and Industrial Research, Osaka University, 8-1 Mihogaoka, Ibaraki, Osaka 567-0047, Japan; taniguti@sanken.osaka-u.ac.jp; 4 Unitech Co., Ltd., Kashiwa 277-0005, Japan; 5 Kyowa-kai Medical Corporation, Osaka 540-0008, Japan; 6 Prophoenix Division, Food and Life-Science Laboratory, Idea Consultants, Inc., Osaka-city, Osaka 559-8519, Japan; 7 Kinshu-kai Medical Corporation, Osaka 558-0041, Japan; 8 Hirotsu Bio Science Inc., Tokyo 107-0062, Japan; 9 Department of Clinical and Molecular Medicine, University of Rome “Sapienza”, Santo Andrea Hospital, via di Grottarossa, 1035-00189 Rome, Italy; andrea.vecchione@uniroma1.it

**Keywords:** exosome, cancer, immunology, surgery

## Abstract

Exosomes (EXs), a type of extracellular vesicles secreted from various cells and especially cancer cells, mesenchymal cells, macrophages and other cells in the tumor microenvironment (TME), are involved in biologically malignant behaviors of cancers. Recent studies have revealed that EXs contain microRNAs on their inside and express proteins and glycolipids on their outsides, every component of which plays a role in the transmission of genetic and/or epigenetic information in cell-to-cell communications. It is also known that miRNAs are involved in the signal transduction. Thus, EXs may be useful for monitoring the TME of tumor tissues and the invasion and metastasis, processes that are associated with patient survival. Because several solid tumors secrete immune checkpoint proteins, including programmed cell death-ligand 1, the EX-mediated mechanisms are suggested to be potent targets for monitoring patients. Therefore, a companion therapeutic approach against cancer metastasis to distant organs is proposed when surgical removal of the primary tumor is performed. However, EXs and immune checkpoint mechanisms in pancreatic cancer are not fully understood, we provide an update on the recent advances in this field and evidence that EXs will be useful for maximizing patient benefit in precision medicine.

## 1. Introduction

Pancreatic cancer is classified as a type of intractable, therapy-resistant cancer, and its overall five-year survival rate has not much changed over the past few decades. Pancreatic cancer is predicted to be the second-leading cause of cancer-related mortality in the next decade in Western countries [1]. Pancreatic cancer is reported to cause tissue invasion and metastasis to distant organs in the early stage of carcinogenesis and during clinical diagnosis, tumors are typically already in the advanced stages [2]. However, several research efforts have focused on the effectiveness of immune therapy combined with surgery, evidence for its use in controlling pancreatic cancer is not enough [3]. Here we update and focus on the recent advances in the field of immuno-surgical therapeutic strategy for pancreatic cancer, which was emerged recently in the relevant of extracellular vesicles (EVs) such as exosomes (EXs) [4].

## 2. Systemic Review of Immune-Surgical Strategies against Pancreatic Cancer

By a systemic review in the PubMed database (https://pubmed.ncbi.nlm.nih.gov), we found that recent publications of both clinical and nonclinical studies by searching keywords “exosome,” “miRNA,” and “pancreatic cancer” have emerged as summarized in Table 1 and Table 2. By noting recent scientific advances in this area, in this study, we focus on the clinical aspects of cancer treatment, especially immune-surgical strategies that monitor the cancer-associated EXs of pancreatic cancer.

## 3. Cellular Exosomes in Pancreatic Cancer

Recent advances in research have resulted in the emergence of precision medicine. However, cancer is a genetic disease, in which tumor-restricting, tumor-suppressor genes and growth-promoting oncogenes are mutated [47], recent research has revealed that cancer cells can actively secrete EVs, including EXs and microvesicles, which are cell-to-cell mediators of metastasis [48].

The involvement of EVs is not limited to cancer cells but extends to other cells of the tumor environment as well, including cancer-associated fibroblasts [49], endothelial cells [50], mesenchymal cells [51], myeloid-derived suppressor cells [52], endothelial progenitor cells [52], a subclass of macrophages [53], antigen-presenting cells [54] and neural cells [55].

Moreover, EVs are reportedly involved in other diseases than cancer, such as modulating metabolic diseases, like type 2 diabetes mellitus [56], amyotrophic lateral sclerosis [57], heart failure [58] and stroke [59]. It is known that these secreted EVs are circulated in peripheral blood, so considerable efforts have focused on the possibility that the clinical examination of EVs may be useful for diagnosing and monitoring human diseases [60].

The EVs are likely involved in cellular signal transduction or cell-to-cell communications in diseases of pancreas. For examples, a previous report showed that c-Met/hepatocyte growth factor receptor and PDL1 expression in circulating EXs in peripheral blood could be used as a diagnostic and prognostic marker for pancreatic cancer [61], but more accurate approaches for disease diagnosis must be developed. Indeed, it has been proposed that, after the surgical removal of primary tumors, immune checkpoint medicine may target marginal invasions in the surrounding tissues and distant organs [62]. Thus, understanding the mechanism of therapy resistance in pancreatic cancer in relation to the tumor microenvironment or immune microenvironment is necessary.

## 4. Bacterial Exosomes in Pancreatic Cancer

Bacterial EVs are studied as a new way to decipher the host–microbiota communications in inflammatory dermatoses [63], colitis [64], intestinal barrier dysfunction [65] and diabetes [66]. However, the precise mechanism remains to be elucidated, the interaction between gut microbes and leaky gut epithelium will increases the uptake of macromolecules like lipopolysaccharide or pro-inflammatory substances from the membranes of microbes leading to chronic inflammation [66]. The recent study indicated that the pancreatic cancer microbiome can promote oncogenesis by induction of innate and adaptive immune suppression, and bacterial ablation was associated with immunogenic reprogramming in pancreatic TME, with a reduction in myeloid-derived suppressor cells and an increase in M1 macrophage differentiation, leading to an efficacy for checkpoint-targeted immunotherapy by upregulating PD-1 expression [67]. However, an involvement of EXs remains to be elucidated, it is suggested that microbiota can promote the crippling immune-suppression characteristic of pancreatic cancer, being a potential therapeutic target of the disease [67].

## 5. Exosomes-mediated Immunity in Pancreatic Cancer

Immunotherapy targeting immune checkpoints has emerged as beneficial for patients with diseases involving the T-cell response system, such as replication-error-prone colorectal [68], esophageal [69] and skin cancers [70]. As a result, the scientific community has become interested in the relevance of immunotherapy in uncharacterized tertiary lymphoid structures [71] as well as various aspects of cell-to-cell communication, including humoral factors, such as cytokines and chemokines [72] and recently in EVs [73]. Given that the original report indicated the involvement of programmed cell death-ligand 1 (PDL1) on tumor cells in the escape from host immune system [74], Some studies have focused in particular on the response to anti-PDL1 therapy [75] and antitumor immunity and memory [76].

It is shown that chemoattractant proteins such as (C–X–C motif) ligand 2 (CXCL2), CXCL8, and CXCL16 were found in cellular EV proteome [77]. The secretome including soluble proteins and extracellular vesicles are highlighted as an acellular regenerative therapy for liver disease [78]. EVs are as expected from the nature of EVs, the recent study indicates that EVs or EV-like nanovesicles will be useful for the potential therapeutic usage as a prospective immunosuppressant, and proposed the possible usage of dual-targeting vesicles, composed of programmed cell death-ligand 1/programmed cell death 1 (PD-L1/PD-1) and cytotoxic T-lymphocyte-associated protein 4/cluster of differentiation 80 (CTLA-4/CD80) [79].

## 6. Exosomes Secretion and Cell-to-Cell Communications in Pancreatic Cancer

EXs, a type of EVs with a diameter of 50–150 nm, are secreted from many cells in health and diseases, including cancer, mesenchymal and immune cells, as mentioned above [49,50,51,53,54,55]. However, DNA has been much less studied as an EV macromolecular component than the others, some publications already mention DNA as an EV/EX component. The previous study demonstrate the importance of future therapeutic potential and correct design of treatment interventions to identify the compartment and mechanisms by which specific DNA, RNA, and proteins are secreted in human disease [80]. It means that the matter is still open for discussion and a different mechanism is already suggested for cell–DNA release. Therefore, it may perhaps be wise not to be so affirmative about dsDNA and EXs, but rather to stress, as suggested, the imperative need for a reassessment of EX/EV composition before any further therapeutic use.

A recent very interesting study studied EXs composition and found important differences with the many published EX cargo compositions, especially with regard to DNA, together with a lack of cytoskeletal elements and glycolysis enzymes [80], suggesting that EX loading is highly regulated process [81], which may be useful in drug delivery for silencing BCR–ABL fusion gene of chronic myelogenous leukemia or for silencing RAD51 and RAD52 [82,83,84]. This finding stresses the imperative needed reassessment of EX/EV composition before any further therapeutic use.

As EX surface contains lipids and proteins derived from cell membranes, and the EX interior contains intracellular substances and biomaterials such as miRNAs, mRNA and proteins, EXs are claimed to be involved in cell-to-cell communication between close and distant cells in various tissues, including cancer cells [85]. miRNAs in EXs are free of Argonaute (Ago) 2 protein, and more than 90% of Ago protein-bounded miRNAs are independent of EVs, suggesting exosomal miRNA is completely independent of miRNA-induced silencing complex (miRISC), and possesses a possible unique function [86,87]. Taken together, EXs secreted from cancer cells have been suggested to be involved in cancer cell survival, malignant transformation, and metastasis and function to favor cancer cells [88].

On the other hand, in the immune system, EXs secreted from some cells function as antigen-presenting vesicles and induce antitumor immunity responses and immune tolerance, which suppress inflammation [89]. In a previous study using state-of-the-art technology for isolation, EXs secreted from cells were demonstrated to mimic somewhat the characteristics of the cells that secrete them and were observed in body fluids, where they have attracted attention as being useful for diagnosing diseases [90]. Diagnosis based on the presence of cancer cells in body fluids and components derived from them is called liquid biopsy, and EX use as diagnostic markers has also been suggested [91].

## 7. Exosomes Carry miRNAs Inside in Pancreatic Cancer

Several biomaterials and metabolic substances are contained inside EXs, including long non-coding RNAs or, the short form, miRNA [85].

### 7.1. miRNAs

**Figure 1:**
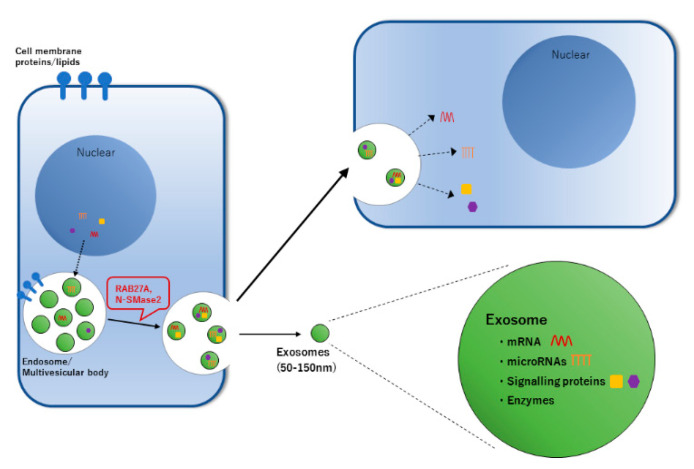
Exosomes can transfer information via cell-to-cell communications. Exosomes (EXs) are membrane vesicles secreted from many kinds of cells. EXs contain various secretory cell-derived proteins and RNA, including endosome-derived proteins, proteins involved in intracellular transport and cell membrane-derived proteins. In addition, they contain lipids derived from the cell membrane of endocrine cells and endosomal membranes. EXs taken up by the target cells fuse with their endosomal membrane to release the contained RNAs into the cytoplasm of the target cells. A released mRNA is translated into a protein, whereas the miRNAs suppress the translation of the target gene and thus EXs control the gene expression in the target cell. In addition, these EXs components are different from those in EX secretory cells. Therefore, a specific mechanism by which EX proteins and mRNA/miRNA are selectively loaded into EXs was suggested.

Because miRNAs inhibit the process of transcription and translation [88], certain miRNAs have been proposed as being involved in the process of gene function regulating growth promotion, chemotherapy resistance, cancer invasion and metastasis, which are useful for diagnosing and monitoring the disease. Extensive efforts have been made to identify miRNAs as therapy-relevant companion diagnostic tools and novel therapeutic targets [89,90,91]. High-speed, next-generation sequencing has facilitated the process of research and development, which indicated that critical functions of miRNAs are dependent on the tissue-specific expression of miRNAs as well as downstream networks. Previous expression analysis allowed for the identification of miR-1246 from gemcitabine-resistant pancreatic cancer cells, with high expression in pancreatic cancer, but abnormal counterparts and subsequent analysis using expression profiling of pancreatic cancer cells demonstrated that cyclin G2 [92] is the target of miR-1246 in the downstream networks [93]. Moreover, recent studies indicated that miR-1246 is involved in tumor immunity by reprogramming macrophages to tumor-supporting macrophages via exosomal miR-1246 in mutant p53 cancers [94]. Interestingly, a methyltransferase of RNAs, METTL3, was shown to promote metastasis of colorectal cancer via the miR-1246/SPRED2/MAPK signaling pathway [95]. However, the relevance of this network in pancreatic cancer has not yet been demonstrated, despite the critical role that METTL3 plays in pancreatic cancer [96]. Accordingly, the precise profiling of single-cell-level approaches would be able to identify the cell-to-cell communications in the tumor microenvironment and truly useful bona fide biomarkers (Figure 1). Moreover, the previous original report indicated that miRNAs silencing by the attachment of peptide nucleotide acid antimiRs to a peptide provided a novel construct that could target tumor microenvironments, which is effectively inhibit especially the miR-155, suggesting broad impacts on the field of targeted drug delivery [97].

### 7.2. Measurements of Epigenetic Information

Epigenetic information contained in DNA exclusively includes the methylation of cytosine at the 5′ position (5mC), which exerts the control function of the downstream gene expression in the promoter and enhancer levels [98]. Nevertheless, epigenetic information in RNA was elusive until state-of-the-art technology was developed to measure the precise position in the sequence and actual modifications [99]. The application of modified mass spectrometry analysis allowed the identification of methylation information of miRNAs, suggesting its usefulness in biomarker screening in the early phases of pancreatic cancer [100].

## 8. Significance of Exosomal PDL1 in Pancreatic Cancer

EXs are secreted from cells via the endosome and multivesicular body, in which RAB27A [101] and neutral sphingomyelinase 2 (N-SMase2), a phosphoprotein exclusively phosphorylated at the serine residues [102], are involved, the EX surface is naturally considered to be highly relevant to the original cells in terms of the density of phospholipids and proteins, as well as major histocompatibility complex and other antigens [73]. The inhibition of RAB27A and N-SMase2 has demonstrated the importance of surface expression [73].

### 8.1. Exosomal PDL1 in Pancreatic Cancer

A previous study reviewed the importance of exosomal and soluble PDL1, a ligand for PD1 receptor in many solid tumors. However, its role in pancreatic cancer remains to be investigated. A recent study indicated that, although no specific difference was detected between the PDL1 amounts in pancreatic cancer patients and in compared patients with chronic pancreatitis and benign serous cystadenoma of the pancreas, PDL1-positive pancreatic cancer patients had a significantly shorter postoperative survival time, suggesting the usefulness of PDL1 as a marker of prognosis [61].

### 8.2. Immuno-Diagnosis and Companion Diagnostics of Pancreatic Cancer

Although the technology used to detect tumor immunological information by EXs likely needs to be more specific, tumor tissue examination has elucidated the involvement of PD1, PDL1, CD8 and FOXP3 [103,104]. Moreover, PDL1 expression was determined to be a poor prognostic factor in patients with high infiltration of CD8 lymphocytes [105,106].

## 9. Exosomes Express PDL1 Outside

### 9.1. Immune Checkpoints

The immune system activates T cells to distinguish cancerous, infected or foreign cells from normal somatic cells and also activates T cells by fine-tuning the checkpoints to monitor healthy cells and recognize and eliminate unhealthy or xenogeneic cells [107]. Cancer cells use not only regulatory T cells and myeloid-derived suppressor cells but also immune checkpoint molecules for immunization in order to avoid attacks from the immune system [108]. The suppressive function is also actively used to escape the immune system regulation. Many cancer cells have a mechanism whereby they are not detected by the immune system and thus grow uncontrolled. For example, some cancers express surface ligands, such as PDL1, that bind to T cells and suppress their activity, allowing them to avoid detection by the immune system [107].

### 9.2. PD1 and PDL1

Whereas PD1 is a receptor that belongs to the CD28 family, which is expressed on activated T cells and myeloid cells, and is also an immune checkpoint molecule, PDL1 is an immune checkpoint protein that acts as a co-suppressor, which suppresses or arrests T-cell responses, and is usually expressed on the surface of antigen-presenting cells [107].

When PDL1 binds to PD1, cytokine production from T cells is reduced, and signals suppressing T-cell activity are transmitted. Tumor cells use this immune checkpoint signaling to escape recognition from T cells in tumors as well as infectious diseases [109]. PDL1 is also strongly expressed on the cell surface of tumor cells and non-transformed cells present in the tumor microenvironment [110]. Activity is suppressed when PDL1 binds to PD1 on the surface of activated cytotoxic T cells. The inactivated T cells then remain in the tumor microenvironment without migrating. Such PD1/PDL1-mediated mechanisms manage the resistance of tumor cells to tumor immunity. Clinical studies on the administration of anti-PD1/PDL1 antibodies [111,112] are investigating whether cancer immunotherapy can reduce such resistance to tumor immunity and maintain the immune response to the tumor.

**Figure 2:**
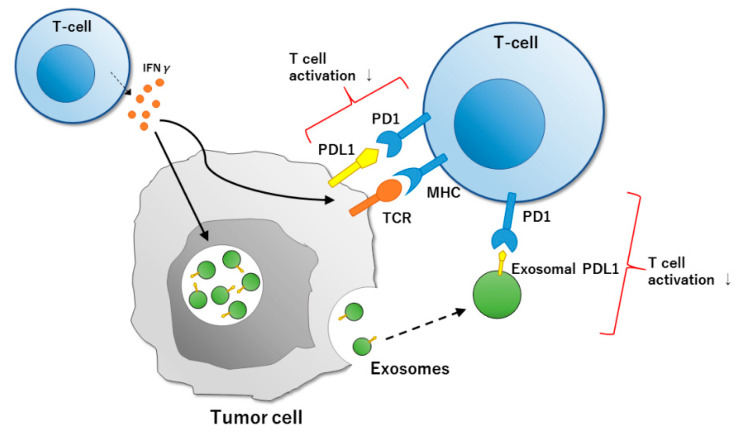
Exosome-mediated PD1 and PDL1 pathways. Interferon-γ (IFNγ) produced by T cells upregulates PDL1 expression in many tumor cells and stimulates endosomes or the multivesicular body to secrete extracellular vesicles and exosomes. When PD1 expressed on activated T cells binds to PDL1 expressed on cancer cells or antigen-presenting cells, T-cell activation is suppressed, and immune escape of cancer cells occurs in the tumor microenvironment.

The study of EXs in solid tumors, such as prostate cancer, has indicated that cancer cells express exosomal PDL1, which interacts with PDL molecules on T cells, resulting in the exhaustion of the T-cell response [76]. Exosomal PDL1 was shown to be induced by the tumor microenvironment [75]. Exposure of interferon gamma from T cells stimulates the expression of PDL1 molecules and their secretion on EXs in cancer cells. But, how the tumor microenvironment (i.e., hypoxia, low nutrition, abnormal vasculature, epithelial–mesenchymal condition) is involved in PDL1 expression and which factors play a role in the cleavage and secretion of the soluble or extracellular form of PDL1 molecules remain to be fully understood. However, the development of a sophisticated centrifuge-based EV separation allows now to precisely analyze EVs, EXs, and the soluble and extracellular forms of proteins [73] Figure 2). Further studies characterizing the tumor microenvironment are needed to maximize the antitumor effects of immune checkpoint inhibitors [113] and to develop new technologies to antagonize exosomal PDL1 in immunotherapy-resistant tumors of the lung [114], breast [115], stomach [116] and head and neck [117], as well as in noncancerous conditions such as periodontitis [118].

## 10. Maximized Surgical Outcome by Immune Strategy against Pancreatic Cancer

To overcome the lethality of pancreatic cancer, the unmet medical needs include (1) detection and diagnosis of early stages of the tumor, (2) elucidation of how this tumor adapts the escape mechanism(s) from immune surveillance and (3) further study of the mechanism that is exploited therapeutically in combination with immune checkpoint inhibitors, such as PD1, PDL1 and CTLA4. Nucleotide sequencing and modification detection of exosomal miRNAs will be beneficial in liquid biopsy during the diagnosis of early stages of pancreatic cancer [100,119]. In addition, monitoring exosomal RNAs will be useful for the early detection of recurrence or metastasis of tumors. In addition, measurement of the EX particle surface will provide such diagnostic information as blood- or urine-based biomarkers, although state-of-the-art technologies have only recently emerged [120]. Moreover, studies using surveillance of malignant transformations in tumors [121] and non-cancerous conditions of the pancreas [122] have shown that the resident memory T (Trem) cells play a role in maintaining tissue homeostasis. Given that transforming growth factor-β (TGF-β) is involved in Trem cell activation [123] and exosomal PDL1 secretion [73], it is likely that a metastasis-prone condition such as the induction of the epithelial-to-mesenchymal transition or the tumor-nested condition is formed in the tumor microenvironment elicited by TGF-β [124], though this mechanism is not yet fully understood. Furthermore, exosomal PDL1 may be involved in the expression of the robustness of the tumor microenvironment. This mechanism is exploited therapeutically after the surgical removal of primary tumors and is modulated by nodal involvement, such as tertiary lymphoid nodal structures [71]. However, further studies in pancreatic cancer are necessary.

## 11. Conclusions

Given that pancreatic cancer is still associated with a very poor prognosis and is resistant to chemo–radiation therapy and because the morbidity rank is increasing especially in the Western world, much emphasis should be placed on the research about the development steps of pancreatic cancer. Recent advances in these fields include an increased understanding of EX biology, development of improved measurement methods for miRNAs early diagnosis techniques, comprehensive integration of knowledge of the tumor immune microenvironments and further development of an efficient strategy of combination therapies. The application of cancer EX monitoring into the immuno-surgical strategy of pancreatic cancer is plausible for use in precision medicine in a near future.

## Figures and Tables

**Table 1:** Nonclinical study of exosomes and miRNAs in pancreatic cancer.

Cells, Pathway, System and Involved Molecules	Characterization of Exosomes	Characterization of miRNAs (Functions)	References
TLR4, dendritic cells	Exosomes isolated	miR-203	[5]
		(miR-203 downregulates TLR4 and downstream cytokines in dendritic cells)	
Regulatory factor X-associated protein (RFXAP)	Exosomes isolated	miR-212-3p	[6]
		(miR-212-3p inhibits RFXAP)	
CD44v6, Tspan8, EpCAM, MET and CD104, a panel of protein and miRNA	NSW	miR-1246, miR-4644, miR-3976 and miR-4306	[7]
		(These miRNAs significantly upregulate pancreatic cancer serum exosomes)	
Macrophage (J771.A1)	Transfection experiment	miR-155 and miR-125b2	[8]
		(miR-155 or miR-125b-2 can achieve stable expression of the microRNAs and these modified tumor-derived exosomes can result in macrophages reprogramming in pancreatic tumor microenvironment)	
Cancer-associated fibroblasts, effect of gemcitabine	Studied by exosome release inhibitor, GW4869	NSW	[9]
ROS, DCK and gemcitabine resistance	Conditioned medium	miR-155	[10]
		(miR-155 downregulates DCK and the functional suppression of miR-155 led to marked abrogation of Gemcitabine chemoresistance)	
Stellate cell-derived exosomes	Conditioned medium; suppressed by GW4869	miR-21-5p and miR-451a	[11]
		(Pancreatic stellate cell-derived exosomes contained a variety of microRNAs such as miR-451a, miR-21-5p)	
C2C12 myotube, insulin resistance, PI3 K/Akt/FoxO1 pathway	Conditioned medium	miRNAs suggested	[12]
SMAD4	Exosomes isolated	miR-494-3p and miR-1260a	[13]
		(miR-494-3p and has-miR-1260a are potential mediators of SMAD4-associated de-regulated calcium fluxes, and create an immunosuppressive myeloid cell background)	
M2 macrophages, PTEN/PI3K	NSW	miR-301a-3p	[14]
		(miR301a-39 induced the M2 polarization of macrophages via activation of the PTEN/PI3Kγ signaling pathway and promote malignant behaviors of pancreatic cancer cells)	
Tumor-associated macrophage, gemcitabine resistance	Rab27 a/b deficient mice	miR-365	[15]
		(Macrophage-derived exosomes as key regulators of gemcitabine resistance in PDAC and demonstrate that blocking miR-365 can potentiate gemcitabine response)	
GIP, GLP-1, PCSK1/PCSK3	Animal model	miR-6796-3p, miR-6763-5p, miR-4750-3p, and miR-197-3p	[16]
		(These miRNAs attenuate the synthesis of GIP and GLP-1 from STC-1 cells, and suppress the expression of PCSK1/3, which is responsible for the post-translational processing of Gip and proglucagon)	
TGF-β	Serum	(467 miRNAs, including 7 overexpressed and 460 underexpressed miRNAs)	[17]
Proof-of-concept study in mice, preclinical animal model	Using magnetic nanopore	11 miRNAs	[18]
		(A panel of extracellular vesicle may be miRNA blood-based biomarkers that can detect pancreatic cancer at a precancerous stage)	
Pancreatic stellate cells (PSCs), ACTA2	Conditioned medium	miR-1246 and miR-1290	[19]
		(Pancreatic cancer cells increase the expression of miR-1246 and miR-1290 in PSCs. Overexpression of miR-1290 induces the expression of ACTA2 and fibrosis-related genes in PSCs)	
Cancer-initiating cells, CD44v6 and Tspan8, reprogramming	Knockdown experiments	NSW	[20]
Cancer-associated fibroblasts, TP53INP1	Conditioned medium	miR-106b	[21]
		(miR-106b promotes GEM resistance of cancer cells by directly targeting TP53INP1)	
AMAD9, bone marrow mesenchymal stem cells	Cocultured	miR-126-3p	[22]
		(miR-126-3p was observed to suppress pancreatic cancer through downregulating ADAM9)	
ZNF689	Conditioned medium	miR-339-5p	[23]
		(miR-339-5p suppresses the invasion and migration of pancreatic cancer cells via direct regulation of ZNF689)	
RNU2-1 in spliceosome	Conditioned medium	miR-1246	[24]
		(miR-1246 is considered an oncomiR in various cancer types. Exosome miR-1246 is derived from RNU2-1 degradation through a non-canonical microRNA biogenesis process)	
Bone marrow mesenchymal stem cells	Exosomes isolated	miR-1231	[25]
		(The exosomes extracted from bone marrow mesenchymal stem cells with high level of miR-1231 inhibit the activity of pancreatic cancer)	
TGF-BR3-mediated TGF-β signaling, tumor-associated macrophage	Exosomes isolated	miR-501-3p	[26]
		(M2 macrophage-derived exosomal miR-501-3p inhibits tumor suppressor TGFBR3 gene and facilitates the development of PDAC by activating the TGF-β signaling pathway, which provides novel targets for the molecular treatment of PDAC)	
Cancer stem cells, gemcitabine resistance	Exosomes isolated	miR-210	[27]
		(Exosomes derived from GEM-resistant pancreatic cancer stem cells mediate the horizontal transfer of drug-resistant traits to GEM-sensitive pancreatic cancer cells by delivering miR-210)	
Dying tumor cells, radiotherapy	Exosomes isolated	miR-194-5p	[28]
		(Exosomal miR-194-5p enhanced DNA damage response in tumor repopulating cells to potentiate tumor repopulation)	

NSW—not studied well; miR—miRNA; PDAC—pancreatic ductal adenocarcinoma; GEM—gemcitabine.

**Table 2:** Clinical significance of exosomes and miRNAs in pancreatic cancer.

Cells, Pathway, System and Involved Molecules	Clinical Endpoints, Merits and Comments	Characterization of Exosomes	Characterization of miRNAs (Functions)	References
Salivary exosome	12 patients and 13 controls	Exosomes isolated	miR-1246 and miR-4644	[29]
			(miR-1246 and miR-4644 in salivary exosomes could be candidate biomarkers for pancreatobiliary tract cancer)	
Plasma	Stage I–IIA, n = 15; healthy subjects (n = 15); diagnosis of localized pancreatic cancer	NSW	miR-196a and miR-1246	[30]
			(miR-196a and miR-1246 are highly enriched in pancreatic cancer exosomes and elevated in plasma exosomes of patients with localized pancreatic cancer)	
Circulating exosomes are superior to exosomal glypican-1	29 cases studied for diagnosis	Exosomal miR studied	High miR-10b, miR-21, miR-30c and miR-181a; low miR-let7a	[31]
			(High exosomal levels of miR-10b, miR-21, miR-30c and miR-181a and low miR-let7a readily differentiate PDAC from normal control and chronic pancreatitis samples)	
Gemcitabine resistance	A cohort	Exosomes isolated	miR-155	[32]
			(The increase of miR-155 induced exosome secretion and chemoresistance ability via facilitating the anti-apoptotic activity)	
Biomarker	16 pancreatic cancer, 18 pancreatitis patients and 20 controls	Exosomes isolated from serum	miR-23b-3p	[33]
			(Overexpression of miR-23b-3p promoted proliferation, migration and invasion capability of pancreatic cancer cells in vitro)	
Tumor-associated stroma	A cohort	Exosomes isolated	miR-145	[34]
			(miR-145-5p exerts an antitumor role in PDAC)	
Circular RNA (circ-RNA), MACC/MET/ERK or AKT pathways	A cohort	Plasma	Circ-PDE8A acting as a ceRNA for miR-338	[35]
p53, TGF-β	Training (40 tumors; 40 controls), testing (112; 116), external validation (41; 50)	Plasma	miR-122-5p, miR-125b-5p, miR-192-5p, miR-193b-3p, miR-221-3p, and miR-27b-3p	[36]
			(These miRNAs may involve in several molecular pathways closely related with p53 signaling pathway, TGF-beta signaling pathway, etc. These miRNAs could act as a non-invasive biomarker in diagnosis and prognosis of pancreatic cancer.)	
p27	A cohort	NSW	miR-222	[37]
			(Tumor-generated exosomes could promote invasion and proliferation of neighboring tumor cells via miR-222 transmission)	
miR-196b, LCN2 and TIMP1	Familial pancreatic cancer	NSW	miR-196b	[38]
			(The combination miR-196b/LCN2/TIMP1 may be a promising biomarker set for the detection of high-grade PDAC precursor lesions in individuals at risk of familial pancreatic cancer families)	
A set of three miRs	32 patients, 29 IPMN, 22 controls	Serum	miR-191, miR-21 and miR-451a	[39]
			(The level of three miRNAs enclosed in serum exosomes can serve as early diagnostic and progression markers of pancreatic cancer and IPMN and considered more useful markers than the circulating miRs)	
Minimally invasive biomarker	Identified in 6 patients and validated in 50 patients	Plasma	miR-451a	[40]
			(Exosomal miR-451a levels may be a useful minimally invasive biomarker for the prediction of recurrence and prognosis in PDAC patients)	
Panel diagnosis by six miRs	A cohort of 30 cancer and 30 controls	Serum	let-7b-5p, miR-192-5p, miR-19a-3p, miR-19b-3p, miR-223-3p and miR-25-3p	[41]
			(These six-miRNA panel in the serum for pancreatic cancer may lead to early and noninvasive diagnosis)	
Pancreatic juice samples, CD63, CD81 and TSG101	27 patients and 8 controls	Exosomes isolated	miR-21 and miR-155	[42]
			(Exosomal miRNAs, including ex-miR-21 and ex-miR-155, in pancreatic juice may be developed as biomarkers for PDAC)	
miRs in portal vein blood (PVB)	55 patients	Exosomes isolated	miR-4525, miR-451a and miR-21	[43]
			(miR-4525, miR-451a and miR-21 in PVB are potential biomarkers identifying patients at high-risk for recurrence and poor survival in resected PDAC patients)	
Epithelial-to-mesenchymal transition	Cancer Genome Atlas (TCGA) data set and a cohort	Exosomes isolated from serum	miR-196b-3p and miR-204-3p	[44]
			(Serum exo-miRNA biomarkers (miR-196b-3p and miR-204-3p) potentially identify the pancreatic tumor status through less-invasive methods)	
Urine exosomes	A cohort	Exosomes isolated	miR-3940-5p/miR-8069 Ratio	[45]
			(The miR-3940-5p/miR-8069 ratio in urine exosomes may be useful as a tool for the diagnosis of PDAC, particularly when used in combination with CA19-9)	
Biomarker for the early diagnosis, nanoparticle biochip	36 patients and 65 controls	Exosomes isolated from plasma	miR-21	[46]
			(Evaluating exosomal miR-21 using the tethered cationic lipoplex nanoparticle biochip may be a useful non-invasive strategy for diagnosing early stage pancreatic cancer)	

NSW—not studied well; miR—miRNA; PDAC—pancreatic ductal adenocarcinoma.
